# Comorbid Depression and Psychosis in Parkinson’s Disease: A Report of 62,783 Hospitalizations in the United States

**DOI:** 10.7759/cureus.5227

**Published:** 2019-07-24

**Authors:** Sundus Imran, Rikinkumar S Patel, Henry K Onyeaka, Muhammad Tahir, Sowmya Madireddy, Pranita Mainali, Sadaf Hossain, Wahida Rashid, Uwandu Queeneth, Naveed Ahmad

**Affiliations:** 1 Neurology, Indiana University School of Medicine, Indianapolis, USA; 2 Psychiatry, Griffin Memorial Hospital, Norman, USA; 3 Epidemiology, Harvard School of Public Health, Boston, USA; 4 Internal Medicine, Penn Medicine Lancaster General Hospital, Lancaster, USA; 5 Internal Medicine, Mamata Medical College, Khammam, IND; 6 Psychiatry, California Instititute of Behavioral Neurosciences and Psychology, Fairfield, USA; 7 Psychiatry, Brookdale Hospital, Brooklyn, USA; 8 Internal Medicine, Dhaka Medical College, Dhaka, BGD; 9 Medicine, Maastricht University, Maastricht, NLD; 10 Psychiatry, University of Texas, Houston, USA

**Keywords:** parkinson’s disease, depression, psychosis, hospitalizations, outcomes, morbidity, dbs, length of stay, cost, demographics

## Abstract

Background

Depression and psychosis are common comorbidities that significantly affects the quality of life and disease outcomes in Parkinson’s disease (PD) patients.

Objective

The aim of this study was to analyze and discern the differences in the hospitalization outcomes, comorbidities, and utilization of deep brain stimulation (DBS) in PD patients with comorbid depression and comorbid psychosis.

Methods

We used the Nationwide Inpatient Sample (2010-2014) and identified PD as a primary diagnosis (N = 62,783), and depression (N = 11,358) and psychosis (N = 2,475) as co-diagnosis using the International Classification of Diseases, Ninth Revision (ICD-9) codes. Pearson’s chi-square test and independent-sample *t*-test were used for categorical data and continuous data, respectively.

Results

White male, older age, and comorbid psychosis were significantly associated with higher odds of having major severity of illness in PD inpatients. The mean length of stay (LOS) was higher in PD patients with psychosis compared to PD with depression (7.32 days vs. 4.23 days; *P *< 0.001), though the mean total charges of hospitalization were lower in psychosis ($31,240 vs. $38,581; *P* < 0.001). Utilization of DBS was lower in PD patients with psychosis versus with depression (3.9% vs. 24.3%; *P *< 0.001).

Conclusion

Psychiatric comorbidities are prevalent in PD patients and are associated with more disease severity, impaired quality of life, and increased use of healthcare resources (higher LOS and cost). They should be considered an integral part of the disease, and a multidisciplinary approach to managing this disease is crucial to improve the health-related quality of life of PD patients.

## Introduction

Parkinson’s disease (PD) is a progressive neurodegenerative disorder that destroys dopamine-producing neurons and results in a movement disorder with motor and non-motor symptoms. It is estimated that about 930,000 people will have PD by 2020 and a possible projection of 1.2 million people by 2030 [1]. These numbers are as alarming as the economic burden estimated at 14.4 billion dollars in 2010, approximately $8.1 billion higher than in non-PD individuals [2]. Not only does PD manifest as motor symptoms, but its non-motor symptoms reflect the advanced stage of the disease of which neuropsychiatric symptoms were the strongest predictor of nursing home admissions [3].

Multiple studies have been done on psychiatric comorbidities in PD patients. Some risk factors for depression in PD patients are severity and duration of illness, presence of tremors, bradykinesia or motor disability, and associated anxiety or psychosis [4]. On the other hand, psychosis is not a primary symptom in PD patients, but its risk increases with comorbid depression, dementia, and longer duration of illness [4]. Depression in PD patients is also underdiagnosed and negatively impacts the course of the disease [5]. PD with a higher prevalence of psychiatric comorbidities is associated with poor health-related quality of life and higher treatment costs [4,6]. The clear pathophysiology behind psychiatric comorbidities and PD remains unclear, but prompt diagnosis and treatment can cure these conditions [7].

We conducted a national study using the nationwide inpatient sample (NIS) data to evaluate 1) differences in demographics and hospital outcomes by psychiatric comorbidities (depression and psychosis) and 2) impact of psychiatric comorbidities on the severity of illness and adverse discharges to skilled nursing facilities or intermediate nursing facilities (SNF/INF).

## Materials and methods

Data source

A retrospective study was conducted using the Healthcare Cost and Utilization Project's (HCUP) NIS data from 2010 to 2014 [8]. The NIS database is considered to determine patterns in demographics and hospital outcomes. It is the largest inpatient database which covers 4,411 hospitals from 45 states in the United States [8]. To protect the privacy of individual patients, physicians, and hospitals, the state and hospital identifiers are de-identified [8]. We were not required to obtain an institutional review board permission to conduct a study on publicly available de-identified data.

Inclusion criteria

We included adult patients (age ≥40 years) with a principal diagnosis of PD based on the international classification of diseases, ninth revision (ICD-9) diagnosis code 332.0 [6]. These sample populations (N = 62,783) were grouped by the presence of comorbid depression (N = 11,358), psychosis (N = 2,475), and non-depression/psychosis group (N = 48950). The ICD-9 diagnoses codes were used to identify co-diagnoses of depression (300.4, 301.12, 309.00, 309.1, or 311) and psychosis (295.00-298.9, 299.10, or 299.11) [6].

Variables of interest

The demographic variables included in this study were age, sex, race, and median household income [9]. To measure the differences in-hospital outcomes in PD patients by the presence of depression and psychosis, the following variables were included: severity of illness that measures the loss of functions, length of stay (LOS), total charges, and utilization of deep brain stimulation (DBS, ICD-9 procedure code 02.93) [9]. In the NIS, LOS means the number of nights the patient remained in the hospital for the principal diagnosis. Total charges during PD hospitalization do not include professional fees and non-covered charges [9].

Statistical analyses

We used descriptive statistics and linear-by-linear association test for categorical data and independent sample *t*-test for the continuous data to measure the differences in demographics and hospital outcomes. We used discharge weight, which is given in the NIS, to obtain a national representation of the sample population [9]. Differences in comorbidities were quantified using chi-square tests. We used a binomial logistic regression model to evaluate the odds ratio (OR) and 95% confidence interval (CI) for major or extreme severity of illness in PD inpatients. The regression model was adjusted for age, sex, race, and median household income. A *P*-value <0.05 was used to determine the statistical significance of the test result. All statistical analyses were done using statistical package for the social sciences (SPSS) version 25 (IBM Corp., Armonk, New York).

## Results

We analyzed a total of 62,783 PD inpatients. Out of these, the prevalence of comorbid depression and psychosis was 18.1% and 3.9%, respectively. Majority of total inpatients were elderly patients (60-79 years, 59.2%). As compared to total inpatients (37.2% females), depression and psychosis were prevalent in females (44.9% and 41.8%). White constituted about four-fifths of the total PD inpatients, and compared to the total, rate of PD with depression was higher in White and PD with psychosis was higher in Black. Also, a higher proportion of PD with psychosis inpatients was from low-income families below 25th percentile compared to PD with depression and total inpatients (28.6% vs. 21.9% and 22.4%, respectively), as shown in Table 1.

**Table 1:** Characteristics of Parkinson’s disease inpatients by psychiatric comorbidities

Variable	Depression (%)	Psychosis (%)	Total (%)	P-value
Inpatients	11358	2475	62783	-
Age at admission, in years				
Mean age (Standard deviation)	71.3 (10.42)	70.1 (10.51)	72.2 (10.66)	<0.001
40 – 59	14.3	15.3	12.9	<0.001
60 – 79	62.2	65.2	59.2	
+80	23.6	19.5	27.9	
Sex				
Male	55.1	58.2	62.9	<0.001
Female	44.9	41.8	37.2	
Race				
White	84.9	76.0	80.6	<0.001
Black	4.3	8.7	6.5	
Hispanic	6.4	7.8	6.8	
Other	4.4	7.5	6.1	
Median household income, in percentile				
0–25^th^	21.9	28.6	22.4	<0.001
26^th^–50^th^	25.5	23.9	25.2	
51^st^–75^th^	26.6	24.2	25.8	
76^th^–100^th^	26.0	23.3	26.6	
Severity of illness, in loss of function				
Minor	32.1	21.2	33.8	<0.001
Moderate	47.7	52.6	44.2	
Major	20.2	26.2	22.0	
Deep brain stimulation (DBS) utilization	24.3	3.9	26.1	<0.001

Moderate to major severity of illness and loss of function was seen in a higher proportion of PD with psychosis inpatients, as shown in Table 1. With advancing age, the odd of association increases with older age patients (>80 years) having 5.3-folds higher odds of major severity of illness (95% CI, 4.830 to 5.840). Male has a marginally higher odds for major severity compared to females. Hispanics are 1.65-fold higher odds (95% CI, 1.528 to 1.781) for major severity compared to Whites, as shown in Table 2.

**Table 2:** Odds of association for major severity of illness in Parkinson’s disease inpatients

Variable	Odds Ratio	95% confidence interval		P-value
		Lower	Upper	
Age at admission, in years				
40–59	Reference			
60–79	2.51	2.290	2.747	<0.001
+80	5.31	4.830	5.840	<0.001
Sex				
Male	1.19	1.138	1.240	<0.001
Female	Reference			
Race				
White	Reference			
Black	1.65	1.528	1.781	<0.001
Hispanic	1.04	0.952	1.128	0.406
Other	0.99	0.909	1.085	0.881
Median household income, in percentile				
0–25^th^	Reference			
26^th^–50^th^	0.98	0.925	1.042	0.547
51^st^–75^th^	1.00	0.946	1.065	0.904
76^th^–100^th^	1.06	0.995	1.118	0.075
Psychiatric comorbidities				
Depression	0.96	0.91	1.01	0.145
Psychosis	1.38	1.252	1.524	<0.001

The logistic regression model was controlled for demographic confounders, and we found a statistically significant higher odds of association of 1.4-fold (95% CI, 1.252 to 1.524) between major severity of illness in PD inpatients and comorbid psychosis.

The utilization of DBS was very low in psychosis inpatients compared to PD with depression and total inpatients (3.9% vs. 24.3% and 26.1%, respectively). PD with psychosis inpatients had a statistically significant (*P* <0.001) longer mean LOS of 7.3 days (±15.33) compared to total (4.1 days ± 8.7) and PD with depression (4.2 days ± 9.83). However, these inpatients with psychosis had a statistically significant (*P* <0.001) lower mean total charges ($31,240 ± 36,926) during PD hospitalization compared to total inpatients ($39,688 ± 43,902) and PD with depression ($38,581 ± 40,137).

## Discussion

We found that comorbid depression (18.1%) was more prevalent among hospitalized PD patients compared to comorbid psychosis (3.9%). Majority of the PD inpatients were males (62.9%) and Whites (80.6%) and had a moderate loss of bodily functions (44.2%). Major severity of illness in PD inpatients was seen in patients with comorbid psychosis (1.4 times higher) with significantly longer mean LOS (7.32 days) compared to those with comorbid depression (4.23 days). DBS utilization was significantly higher in PD patients with comorbid depression (24.3%) versus comorbid psychosis (3.9%).

Psychiatric illnesses are prevalent in PD and this may significantly impact the patient’s morbidity and quality of life [10-12]. Several studies have established the association between depression and PD [13-15]. In our cross-sectional study, we found that comorbid depression is prevalent (18.1%) among hospitalized patients with PD. This correlates with previous literature showing a high prevalence of comorbid depression among adults with PD. In a systematic review and meta-analysis conducted by Goodarzi et al., the incidence of depression among PD patients was reported to be 22.9% [15]. The prevalence of psychosis in PD has been challenging to determine, and many past studies indicate varying results. In a longitudinal survey of Forsaa et al., 230 PD patients were followed for twelve years, and 60% had reported psychotic symptoms [16]. On the contrary, a cross-sectional study involving 236 PD patients found that 13% reported psychotic symptoms [17].

PD is a neurodegenerative disease, and its incidence increases with age with advanced age being a risk factor for developing PD [18]. Fifty-nine percent PD inpatients were elder patients (60-79 years) and compared to the overall total, a higher rate of comorbid depression (62.2%) and psychosis (65.2%) was seen in this age group. Elder PD patients had a higher odd (2.5 times in 60-79 years, and 5.3 times in +80 years) of major severity of illness with loss of bodily functions compared to middle-age (40-59 years) patients. Given the progressive nature of the disease, and advancing age associated with comorbidities, and poor health outcomes, including mortality, maybe the reason for worsening of functionality in older age PD patients.

The prevalence of PD was higher in males (62.9%), which was also seen in past studies [18-20]. Shulman et al. found that the incidence of PD is lower in postmenopausal women and postulate that estrogen may play a protective role [21]. Compared to the overall total (37.2%), comorbid depression (44.9%) and psychosis (41.8%) was seen at a higher rate in females [22-24]. We found that men with PD have a marginally higher risk of major severity of illness compared to females. Lubomski et al. reported that men have a lower quality of life in activities of daily living (ADL), cognition, and communication sub-scales [24].

On the contrary, Georgiev et al. found that women with PD show better cognitive abilities and outperform men in verbal cognitive tasks but show more pain symptoms, and experience more depression [25]. The hospitalized Black PD patients had 1.7-fold higher odds of major severity of illness compared to the White with PD. The racial differences in mortality and disease incidence among PD patients exist [26-27]. Willis et al. found that Black patients with PD had a higher likelihood of death during the six-year study period compared to Whites with PD [27]. Our study is the first to highlight the racial difference in terms of the severity of illness among hospitalized PD patients in the US.

Furthermore, we observed that the LOS and total charges of hospitalization for PD differs among comorbidities (depression and psychosis). PD patients with comorbid psychosis had a longer mean LOS (7.3 days) compared to total PD inpatients (4.0 days) and PD with comorbid depression (4.2 days). Although PD patients with comorbid psychosis had higher hospitalization cost compared to the PD inpatient with psychosis, and this discrepancy may be due to the very low utilization of DBS in PD patients with comorbid psychosis (3.9% vs. 26.1 in total PD inpatients). In the study by Patel et al., DBS utilization, as well as therapeutic procedures, were less commonly performed in PD patients with depression in comparison to those without depression [6].

Our findings contrast with prior studies which found that PD with psychosis resulted in the more extended length of hospitalization and higher costs of care (including inpatient and long-term care) [28-29]. Hermanowicz et al. noted that all expenses were higher for PD patients with comorbid psychosis, with the highest cost differentials found in long-term care, SNF, and inpatient costs ($10,125 in PD with psychosis vs. $6024 in PD without psychosis) [29].

One of the strengths of our study is that we used the NIS database, which is a nationally representative sample of patients diagnosed with PD. The large sample size we obtained ensures that our study is high powered to identify any differences that exist in the subgroups of patients analyzed. Also, our application of sampling weights to generate estimates of inpatient outcomes enables extrapolation and generalizability of our results to a much larger population than the sample studied. Furthermore, this is the first study, to our knowledge, to evaluate and compare the differences in the impact of comorbid psychosis and comorbid depression in PD patients regarding hospital outcomes and morbidity. However, the results in our study should be interpreted with several limitations in mind. Our utilization of inpatient data (and not patient-level data) as the unit of analysis precludes generalizability for all patients with PD. Also, given the nature of the database, we did not account for re-hospitalization. Re-hospitalizations may add to the total disease burden and may help explain some of the differences in hospitalization costs. Furthermore, we did not adjust for other important characteristics such as patients' complications and comorbidities, which may affect our results. Therefore, we recommend that future research should utilize clinical data and employ longitudinal based designs to evaluate differences in outcomes between PD patients with psychosis and those with depression.

## Conclusions

Our study establishes the negative impact of depression and psychosis in PD concerning hospitalization-related outcomes including the illness severity, comorbid conditions, LOS and total charges during hospitalization, and utilization of DBS. The results of our study suggest that healthcare providers should actively screen for psychiatric comorbidities like depression and psychosis in patients with PD. These comorbidities are prevalent in the majority of patients with PD and are associated with more disease severity, impaired quality of life, and increased use of healthcare resources (higher LOS and cost). Psychiatric comorbidities in PD should be considered an integral part of the disease, and a multidisciplinary approach to managing this disease is crucial to improve the overall outcome and the health-related quality of life of PD patients.

## References

[1] Marras C, Beck JC, Bower JH (2018). Prevalence of Parkinson's disease across North America. NPJ Parkinsons Dis.

[2] Kowal SL, Dall TM, Chakrabarti R, Storm MV, Jain A (2013). The current and projected economic burden of Parkinson's disease in the United States. Mov Disord.

[3] Aarsland D, Larsen JP, Tandberg E, Laake K (2000). Predictors of nursing home placement in Parkinson's disease: a population-based, prospective study. J Am Geriatr Soc.

[4] Grover S, Somaiya M, Kumar S, Avasthi A (2015). Psychiatric aspects of Parkinson's disease. J Neurosci Rural Pract.

[5] Dobkin RD, Rubino JT, Friedman J, Allen LA, Gara MA, Menza M (2013). Barriers to mental health care utilization in Parkinson's disease. J Geriatr Psychiatry Neurol.

[6] Patel RS, Makani R, Mansuri Z, Patel U, Desai R, Chopra A (2017). Impact of depression on hospitalization and related outcomes for Parkinson's disease patients: a nationwide inpatient sample-based retrospective study. Cureus.

[7] Marsh L (2013). Depression and Parkinson's disease: current knowledge. Curr Neurol Neurosci Rep.

[8] (2019). Overview of the national (nationwide) inpatient sample (NIS). https://www.hcup-us.ahrq.gov/nisoverview.jsp.

[9] HCUP) HCaUP (2018 (2019). NIS description of data elements. https://www.hcup-us.ahrq.gov/db/nation/nis/nisdde.jsp.

[10] Cooney JW, Stacy M (2016). Neuropsychiatric issues in Parkinson's disease. Curr Neurol Neurosci Rep.

[11] Thanvi BR, Munshi SK, Vijaykumar N, Lo TC (2003). Neuropsychiatric non-motor aspects of Parkinson's disease. Postgrad Med J.

[12] Alvarado-Bolaños A, Cervantes-Arriaga A, Rodríguez-Violante M (2015). Impact of neuropsychiatric symptoms on the quality of life of subjects with Parkinson's disease. J Parkinsons Dis.

[13] Smeltere L, Kuznecovs V, Erts R (2017). Depression and social phobia in essential tremor and Parkinson's disease. Brain Behav.

[14] McDonald WM, Richard IH, DeLong MR (2003). Prevalence, etiology, and treatment of depression in Parkinson's disease. Biol Psychiatry.

[15] Goodarzi Z, Mrklas KJ, Roberts DJ, Jette N, Pringsheim T, Holroyd-Leduc J (2016). Detecting depression in Parkinson disease: a systematic review and meta-analysis. Neurology.

[16] Forsaa EB, Larsen JP, Wentzel-Larsen T, Goetz CG, Stebbins GT, Aarsland D, Alves G (2010). A 12-year population-based study of psychosis in Parkinson disease. Arch Neurol.

[17] Rodriguez-Violante M, Velazquez-Osuna S, Cervantes-Arriaga A, Corona-Vazquez T, de la Fuente-Sandoval C (2015). Prevalence, associated factors and phenomenology of psychosis in patients with Parkinson's disease (PD). Gac Med Mex.

[18] Baldereschi M, Di Carlo A, Rocca WA (2000). Parkinson's disease and parkinsonism in a longitudinal study: two-fold higher incidence in men. ILSA working group. Italian longitudinal study on aging. Neurology.

[19] Kovacs M, Makkos A, Aschermann Z (2016). Impact of sex on the nonmotor symptoms and the health-related quality of life in Parkinson's disease. Parkinsons Dis.

[20] Alves G, Muller B, Herlofson K (2009). Incidence of Parkinson's disease in Norway: the norwegian parkwest study. J Neurol Neurosurg Psychiatry.

[21] Shulman LM (2007). Gender differences in Parkinson's disease. Gend Med.

[22] Solla P, Cannas A, Ibba FC (2012). Gender differences in motor and non-motor symptoms among Sardinian patients with Parkinson's disease. J Neurol Sci.

[23] Martinez-Martin P, Falup Pecurariu C, Odin P (2012). Gender-related differences in the burden of non-motor symptoms in Parkinson's disease. J Neurol.

[24] Lubomski M, Louise Rushworth R, Lee W, Bertram KL, Williams DR (2014). Sex differences in Parkinson's disease. J Clin Neurosci.

[25] Georgiev D, Hamberg K, Hariz M, Forsgren L, Hariz GM (2017). Gender differences in Parkinson's disease: a clinical perspective. Acta Neurol Scand.

[26] Dahodwala N, Siderowf A, Xie M, Noll E, Stern M, Mandell DS (2009). Racial differences in the diagnosis of Parkinson's disease. Mov Disord.

[27] Willis AW, Schootman M, Kung N, Evanoff BA, Perlmutter JS, Racette BA (2012). Predictors of survival in patients with Parkinson disease. Arch Neurol.

[28] Fredericks D, Norton JC, Atchison C, Schoenhaus R, Pill MW (2017). Parkinson's disease and Parkinson's disease psychosis: a perspective on the challenges, treatments, and economic burden. Am J Manag Care.

[29] Hermanowicz N, Edwards K (2015). Parkinson's disease psychosis: symptoms, management, and economic burden. Am J Manag Care.

